# Neuroplasticity and the Biological Role of Brain Derived Neurotrophic Factor in the Pathophysiology and Management of Depression

**DOI:** 10.7759/cureus.11396

**Published:** 2020-11-09

**Authors:** Sumita Chakrapani, Noha Eskander, Lorenzo A De Los Santos, Basiru A Omisore, Jihan A Mostafa

**Affiliations:** 1 Psychiatry, California Institute of Behavioral Neurosciences & Psychology, Fairfield, USA; 2 Cardiothoracic Surgery, Icahn School of Medicine at Mount Sinai, New York, USA; 3 Anesthesiology, California Institute of Behavioral Neurosciences & Psychology, Fairfield, USA; 4 Internal Medicine, Claude Mandel Medical Centre, Chicago, USA; 5 Internal Medicine, California Institute of Behavioral Neurosciences & Psychology, Fairfield, USA; 6 Psychiatry, California Institute of Behavorial Neurosciences & Psychology, Fairfield, USA

**Keywords:** brain-derived neurotrophic factor, depression, neuroplasticity

## Abstract

Depression is a mental illness that can have serious implications if left untreated. Studies involving a neurotrophic factor called brain derived neurotrophic factor (BDNF) and its associated signaling pathways have solidified our understanding of the pathophysiology of depression. The objective of this literature review is to gain a better understanding of the mechanism by which reduced levels of BDNF are implicated in depression and how antidepressants facilitate the treatment of depression by increasing BDNF levels. The specific approach is to learn about the key involvements of BDNF and its receptor TrkB (tropomyosin receptor kinase B) and how their interactions and subsequent intracellular signaling cascades bring about enhanced neuroplastic changes. In this literature review, we searched for past review articles focusing on BDNF. We collected data using PubMed and created a summary of our findings. The results showed that stress and depression through the reduction of BDNF levels contribute to neuroplastic changes while antidepressants through enhanced BDNF levels are able to generate positive neuroplastic outcomes and thereby help resolve depressive symptoms. In this paper, we will delve into how a better understanding of the neural circuitry involving BDNF will enable us to both understand how current antidepressants work in the limbic regions of the brain as well as search for novel rapid-acting antidepressants to use in clinical practice.

## Introduction and background

Depression is a mood disorder that can have profound implications on one's personal as well as professional life. It places a tremendous burden on the economy as it can contribute to rising unemployment rates.

Brain-derived neurotrophic factor (BDNF) is a neurotrophic factor that is pivotal in our understanding of the pathophysiology of depression. Studies have shown that the volume of the hippocampus and prefrontal cortex (PFC) is reduced in patients with depression [1-2]. Reduced levels of BDNF were found in the hippocampus and prefrontal cortex in rodent models after exposure to different types of stress [3-5]. Post-mortem analyses also reveal reduced BDNF levels in these areas in the brains of patients with depression [3]. The discovery that BDNF levels are decreased in the serum of depressed patients and that it can be reversed with antidepressants has important implications for the role of BDNF as a biomarker of depression as well as the quest for adequate treatment [6-7]. Antidepressants have the opposite effect on BDNF when compared to stress and depression - they increase the expression of BDNF in the hippocampus and PFC [3,5]. A lot of research that has been completed to date on BDNF supports the neurotrophic hypothesis of depression. This hypothesis emphasizes that depression is a result of decreased BDNF, which in turn leads to neuroplastic developments such as neuronal loss, decreased hippocampal neurogenesis and loss of glia and that antidepressants increase levels of BDNF, thereby reversing these changes of neuron loss as well as cell loss [3,8]. This neurotrophic hypothesis has been well studied using animal models using overexpression or silencing of the BDNF gene. These studies have shown that BDNF insertion results in antidepressant response in animal models and that BDNF is necessary for the antidepressants to take effect [3,5]. 

There are two key components - BDNF and its receptor tropomyosin receptor kinase B (TrkB) both of which are fundamental not only in understanding the pathophysiology of depression but also in discovering novel therapeutic agents to treat depression. We now know that BDNF is responsible for significant neuroplastic changes that lead to the improvement of depressive symptoms [9].

Most of the antidepressants available can be classified as monoaminergic antidepressants that elevate synaptic levels of serotonin and norepinephrine. Although this increase happens immediately (within a few hours), it takes much longer (weeks) for antidepressants to take effect and elicit an antidepressant effect in patients with depression [10]. One explanation for this time lag is that it takes time for hippocampal neurogenesis to be initiated by antidepressants. Studies in rodents have shown that chronic (not acute) treatment with antidepressants leads to a rise in the number of proliferative cells and new neurons, which occurs around the same time course that antidepressants take effect [11-12]. It can hence be concluded that antidepressants need to be administered chronically to allow the induction of hippocampal neurogenesis to take place which takes time. Studies of post-mortem brains of patients with depression who had been taking antidepressants demonstrated the presence of hippocampal neurogenesis [13-15]. Ablation of neural progenitor cells (NPCs) in the adult hippocampal dentate gyrus (DG) attenuated the antidepressant response in mice, again supporting the idea that hippocampal neurogenesis is a prerequisite for antidepressants to exert their antidepressant behavioral effect [11]. Numerous studies have shed light on BDNF as the key factor involved in antidepressant-induced neuroplastic changes such as hippocampal neurogenesis. It has been demonstrated time and again that BDNF expression is enhanced in the hippocampus after chronic treatment with antidepressants in both rodents and humans [16-18].

## Review

Method

PubMed was searched using MeSH keyword brain-derived neurotrophic factor. Table 1 shows the search results using MeSH keyword brain-derived neurotrophic factor.

**Table 1:** PubMed MeSH keyword search results * Subheadings include administration and dosage, blood, biosynthesis, classification, deficiency, drug effects, pharmacology, physiology, and therapeutic use.

Keyword	Database	Number of results
Brain derived neurotrophic factor	PubMed	23,938
Brain derived neurotrophic factor with subheadings* added to search builder	PubMed	15,188

Results

The PubMed search using keyword "brain derived neurotrophic factor" was modified using additional subheadings including administration and dosage, blood, biosynthesis, classification, deficiency, drug effects, pharmacology, physiology, and therapeutic use. These subheadings were added to the PubMed search builder. This search yielded 15,188 results. Only articles containing full text were included. In addition, only articles involving human studies in the English language were included. This study focused on review articles only. After applying all of these additional criteria, the number of results was 348. After careful consideration of all of these studies, 28 articles were selected to complete this literature review in order to understand the association between BDNF and depression.

Discussion

Several neurotrophic factors have been studied. The one that piques our interest the most, in terms of understanding the pathophysiology of depression, is brain-derived neurotrophic factor (BDNF). Stress and depression cause neuronal structural and morphological changes by lowering BDNF levels. Initially, neurotrophic factors were studied for their involvement in maturation and survival of neurons during brain development. However, now it is known that neurotrophic factors are important in the adult brain. These factors have significant roles in bringing about neuroplastic changes and neuron survival [19]. BDNF is one of the nerve growth factors that are abundant in the brain. Its expression is regulated in several ways - one is at the level of gene expression and the other is in terms of activity-dependent secretion at synapses. 

Stress and depression cause a decrease in the volume of limbic brain regions [1,2]. Attenuated BDNF signaling has been implicated in this reduction of brain volume [20,21]. Post-mortem studies also show a decrease in the neuronal cell body size, deterioration of processes, and the decline of glia in PFC [22-24]. A recent post-mortem analysis of a study involving depressed patients shows a decrease in the number of synapses in the PFC [25]. Neuronal atrophy is defined as a decrease in dendritic arborization in addition to a decrease in the number of spine synapses [26-28]. In addition to the neuronal loss described above, chronic stress is also a factor in diminishing neurogenesis, or the birth of new neurons and glia, in the dentate gyrus (DG) of the hippocampus [29]. Postmortem analysis of depressed patients treated with antidepressants has shown the presence of increased numbers of newborn cells in the hippocampus. This finding is in agreement with preclinical studies which have reported that chronic administration of antidepressants leads to increased neurogenesis in the adult hippocampus [13-15]. These findings in post-mortem analysis of patients with depression and also those treated with antidepressants provide an optimistic outlook for the future treatment of patients afflicted with depression. These studies have enabled us to have a better understanding of how depression affects key structures in the limbic system of the brain, and they have also revealed that treatment with antidepressants does reverse those effects. Antidepressants enable BDNF and its signaling pathways to enhance synaptic plasticity.

The Neurotrophic Hypothesis of Depression

The neurotrophic hypothesis of depression focuses on the pathophysiology of depression. This theory highlights that stress and depression decrease the expression of neurotrophic factors such as BDNF in brain regions including the hippocampus and PFC. On the other hand, antidepressant treatment reverses these changes by increasing the expression of BDNF in the hippocampus and PFC [3,5]. Rodents exposed to various types of physical or social stress are found to have low levels of BDNF in the hippocampus and PFC [3-5]. The positive finding is that the neuronal atrophy and structural and morphological changes brought about by low BDNF levels is reversible with antidepressants [30-32]. Postmortem studies have shown improved BDNF signaling after treatment with antidepressants [18]. Once it was understood that antidepressants bring about a therapeutic response by inducing adult neurogenesis in the hippocampus [13-15], the quest was to determine if BDNF is necessary in order for antidepressants to bring about this change. Investigations with BDNF null mice and mice overexpressing truncated TrkB (TrkB.T1) supported that BDNF is indeed necessary for long-term neuron survival induced by antidepressants [33]. In addition, when antidepressants were administered to mice without TrkB (TrkB is the high-affinity receptor for BDNF) in hippocampal neural progenitor cells (NPCs), there was no evidence of antidepressant-induced proliferation and neurogenesis [34]. Therefore, it can be gathered from these studies that BDNF is a prerequisite for antidepressants to generate their beneficial effects of hippocampal neurogenesis and survival, thereby improving patient response to these medications. The neurotrophic hypothesis of depression has shed light on our comprehension of the intricate neuroplastic changes that occur as a result of BDNF and its receptor and downstream pathways that get activated. Equipped with this knowledge, we can direct our efforts in the future toward looking for BDNF analogs or similar molecules that produce these same neuroplastic changes in the limbic areas of the brain.

Classification of BDNF: A Look at The Molecular Level

BDNF is a growth factor that has been intensively studied in recent years due to its multiple functions in the central nervous system (CNS). It is involved in neuron maturation and various other neuroplastic changes [35]. BDNF is classified under the neurotrophin family which includes nerve growth factor (NGF), neurotrophin-3 (NT3) and neurotrophin-4 (NT 4). The BDNF gene has nine promoters, each of which controls the expression of unique BDNF transcripts that encode the same BDNF protein [36,37]. 

**Figure 1:**
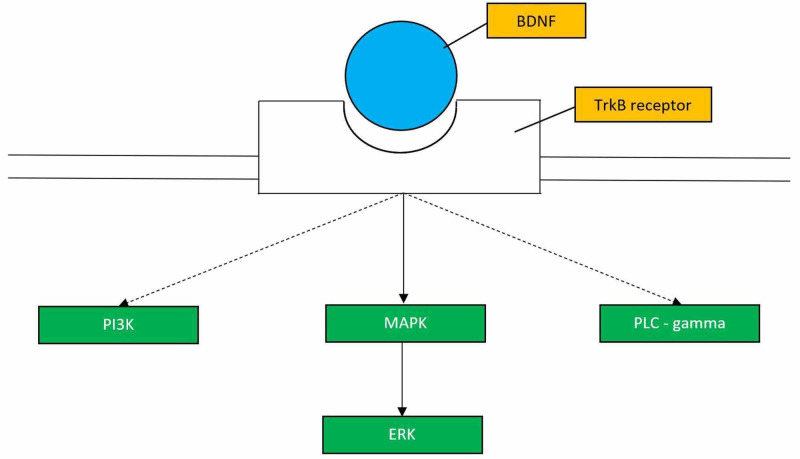
BDNF-TrkB Signaling Pathways This figure (adapted from Figure 3 of [39]) shows the pathways that are activated upon BDNF binding to the TrkB receptor. BDNF: brain-derived neurotrophic factor; TrkB: tropomyosin receptor kinase B; Pi3k: phosphatidylinositol 3-kinase; MAPK: mitogen-activated protein kinase; ERK: extracellular signal-regulated kinase; PLC-gamma: phospholipase C-gamma.

The BDNF gene codes for a precursor peptide, proBDNF. There are a number of intricate steps involved in the production of mature BDNF. It is first made as a precursor protein (preproBDNF) in the endoplasmic reticulum. After the cleavage of the peptide, proBDNF is directed to the Golgi. The proBDNF with the help of intracellular and extracellular proteases is transformed into the mature BDNF. Mature BDNF then binds its high affinity receptor TrkB [38]. TrkB receptor contains tyrosine residues in its kinase domain. TrkB activation initiates multiple intracellular signaling pathways such as mitogen-activated protein kinase/extracellular signal-regulated kinase (MAPK/ERK), phosphatidylinositol 3-kinase (PI3K), and phospholipase C (PLC)-gamma pathways which serve to enhance neurogenesis and synaptic plasticity (Figure 1). When BDNF binds the TrkB receptor, it results in the activation of several downstream signaling cascades that promote the development of favorable neuroplastic phenomena. One must keep in mind that it is critical to design drugs that activate all of the pathways triggered by the BDNF-TrkB receptor interaction in order to reap the beneficial effects of improved synaptic plasticity and synaptic strength. 

A Closer Look at the TrkB Receptor

Studying the TrkB receptor has consolidated our knowledge that BDNF is indeed necessary for antidepressants to exert their therapeutic response. These antidepressants depend on functional TrkB receptors to elicit antidepressant behavior in preclinical models [40,41]. Chronic treatment with antidepressants leads to the activation of TrkB receptor (via phosphorylation) in the hippocampus and anterior cingulate cortex. This event again supports the fact that the BDNF-TrkB interaction in these specific areas of the brain is imperative in eliciting an antidepressant response [41]. This study also concluded that conventional antidepressants depend on monoamines to activate the BDNF signaling pathway since monoamine depletion precludes the activation of TrkB receptor by antidepressants [40]. By understanding the role of the TrkB receptor, it would be beneficial to think along the lines of creating a BDNF agonist with the ability to cross the blood-brain barrier and bind the TrkB receptor, thereby triggering the activation of the pathways mentioned in Figure 1.

Stress, Antidepressants, and Synaptogenesis

Both stress and antidepressant treatment not only play a role in neurogenesis, but they also contribute to the intricate dendritic arbor of neurons. The intrinsic ability of neurons to form spine synapses or synaptogenesis is an important form of neuroplasticity. Synaptogenesis refers to changes that take place an intracellular level in response to synaptic activity, and it is a key mechanism that allows one to process information and then make adaptive changes. Cellular models of learning and memory such as long-term potentiation (LTP) are used to understand how synaptogenesis works. Increased activity in the synapses and increased glutamate transmission result in maturation of spine synapses and spine density (often described as mature “mushroom” spine). This maturation takes place via insertion of glutamate receptors in the postsynaptic membrane [42]. Even minimal restraint stress (as little as 7 days) [27] causes reduction of dendrites and spines in the PFC, and this has been implicated in depressive behaviors after inducing a chronic stressful situation [43]. These studies validate the suggestion that reduction in the intricate dendritic arbor of neurons results in the reduced volume of hippocampus and PFC found in patients with depression. BDNF’s contribution to the dendritic complexity and spine formation has been studied in mutant mice. A gene knockin of BDNF Met polymorphism was created, and studies revealed that having even one copy of this human variant reduces the CA3 apical dendrites in the hippocampus [44]. By studying LTP and understanding the effects on synaptogenesis, we can now appreciate how chronic stress impacts the brain on several different levels including the molecular as well as the cellular level. Only by gaining a better grasp on these models can we begin to determine strategies to curb these changes involving synaptogenesis to curb depressive symptoms.

Implications of Single Nucleotide Polymorphism (Val66Met) in BDNF Gene

Alterations in BDNF expressions can predispose an individual to psychiatric illnesses, including mood disorders and impaired cognition [45]. A single nucleotide polymorphism (SNP) in the BDNF gene [20] (namely in pro-BDNF region) involves a change from valine (val) to methionine (met, i.e., val66met) in codon 66. People with the val66met SNP have impaired episodic memory and abnormal hippocampal function which is thought to be due to problems involving intracellular movement and activity-dependent release of BDNF [20]. Studies have been performed with genetically modified mice carrying the human BDNF val66met alleles. These studies have produced similar results. When exposed to stressful conditions, these mice expressed depressive-like phenotype and also memory deficits. Also, these mice exhibited diminished synaptic plasticity in the hippocampus and medial PFC [46,47]. It was found that giving the antidepressant fluoxetine chronically to mice with val66met SNP in BDNF gene (BDNF met/met) produced no response. It is assumed that this lack of response is because this SNP prevents BDNF secretion [44]. In BDNF met/met mice, both selective serotonin reuptake inhibitor (SSRI) induced enhancement of BDNF levels and synaptic plasticity were reduced [48]. It can be gleaned that the lack of response to SSRIs may be associated with failure to induce synaptic plasticity. These studies have improved our understanding of how antidepressants exert their beneficial effect. This SNP in the BDNF gene in both humans and mice signifies once again that BDNF and the improved synaptic plasticity it brings about is crucial for antidepressants to work. These studies have profound implications for clinical practice in psychiatry. This SNP is the only objective evidence we have when it comes to diagnosing depression. It remains to be discussed whether these genetic analyses such as SNP (Val66Met) in the BDNF gene should possibly be used as an adjunct to support the diagnosis of depression as defined by *Diagnostic and Statistical Manual of Mental Disorders, Fifth Edition *(DSM-5) criteria. It is challenging as physicians to often understand the severity of a patient’s depressive symptoms since its purely subjective based on ambiguous criteria such as anhedonia, feelings of guilt/worthlessness, changes in sleep and appetite [49]. However, providing a concrete means to support these patient-described findings with genetic evidence to back up the diagnosis may prove to be beneficial.

With respect to the research that has been done to date on BDNF, there have been both human studies (including post-mortem analyses and neuroimaging studies) as well as animal model studies. When compared to the animal studies, the human studies have more validity in clinical practice as they provide a better quality of evidence and there is less bias observed in these studies. There are quite a number of limitations to using animal models in the study of depression. It is well recognized that the symptoms of depression are based on subjective features described by patients and it can often be difficult to assess. On a similar note, it is even more difficult to accurately characterize these symptoms in rodent models. The other point to be considered is to what extent the findings observed in rodents can be extrapolated to understand depressive behavior in humans. A lot of the research done on animal models studies the effects of chronic stress leading up to depression. While this method seems like an appealing way to study depression, it can be debated what will qualify as an adequate stressor to conduct the research. This issue is one of the challenges behind conducting animal studies.

Study limitations

One limitation of this study is that specific keywords related to BDNF were searched on PubMed (namely administration and dosage, blood, biosynthesis, classification, deficiency, drug effects, pharmacology, physiology, and therapeutic use). Any topics outside of those mentioned are beyond the scope of this study. In addition, a systematic review of the topic was not completed. Also, a quality assessment of the studies was not performed.

## Conclusions

In this article, we have delineated the key role of BDNF in bringing about neuroplastic changes and its implications in the pathophysiology and management of depression. Stress and depression lead to reduced BDNF levels while antidepressant treatment reverses this by raising BDNF levels. Chronic stress reduces neurogenesis and antidepressants enhance neurogenesis as well as synaptogenesis in the adult hippocampus. The BDNF gene is tightly regulated and undergoes several steps to reach its mature form. Its interaction with the TrkB receptor activates various signaling pathways that play a fundamental role in improved neuroplastic phenomena. SNPs in the BDNF gene can increase an individual’s susceptibility to mood disorders such as depression. With a better understanding of BDNF and its crucial role in neuroplasticity, it is imperative that we direct future research toward designing rapid-acting antidepressants that maximize and utilize the full potential of BDNF’s ability to influence and improve synaptic plasticity. More specifically, we should direct our efforts toward discovering BDNF agonists or agents that activate the TrkB receptor. We need to conduct large scale observational studies as well as randomized clinical trials with large population sizes. Future research should emphasize gene deletions of BDNF and TrkB receptors to unravel the detailed signaling pathways through which BDNF mediates its neuroplastic phenomena.

## References

[1] Drevets W, Price JL, Furey ML (2008). Brain structural and functional abnormalities in mood disorders: implications for neurocircuitry models of depression. Brain Struct Funct.

[2] Macqueen G, Yucel K, Taylor VH, Macdonald K, Joffe R (2008). Posterior hippocampal volumes are associated with remission rates in patients with major depressive disorder. Biol Psychiatry.

[3] Duman R, Monteggia LM (2006). A neurotrophic model for stress-related mood disorders. Biol Psychiatry.

[4] Krishnan V, Nestler EJ (2008). The molecular neurobiology of depression. Nature.

[5] Castren E, Rantamaki T (2010). The role of BDNF and its receptors in depression and antidepressant drug action: reactivation of developmental plasticity. Dev Neurobiol.

[6] Bocchio-Chiavetto L, Bagnardi V, Zanardini R (2010). Serum and plasma BDNF levels in major depression: a replication study and meta-analyses. World J Biol Psychiatry.

[7] Sen S, Duman RS, Sanacora G (2008). Serum brain-derived neurotrophic factor, depression, and antidepressant medications: meta-analyses and implications. Biol Psychiatry.

[8] Duman R, Heninger GR, Nestler EJ (1997). A molecular and cellular theory of depression. Arch Gen Psychiatry.

[9] Björkholm C, Monteggia LM (2016). BDNF - a key transducer of antidepressant effects. Neuropharmacology.

[10] Boku S, Nakagawa S, Toda H, Hishimoto A (2018). Neural basis of major depressive disorder: beyond monoamine hypothesis. Psychiatry Clin Neurosci.

[11] Surget A, Saxe M, Leman S (2008). Drug-dependent requirement of hippocampal neurogenesis in a model of depression and of antidepressant reversal. Biol Psychiatry.

[12] Santarelli L, Saxe M, Gross C (2003). Requirement of hippocampal neurogenesis for the behavioral effects of antidepressants. Science.

[13] Boldrini M, Hen R, Underwood MD, Rosoklija GB, Dwork AJ, Mann JJ, Arango V (2012). Hippocampal angiogenesis and progenitor cell proliferation are increased with antidepressant use in major depression. Biol Psychiatry.

[14] Boldrini M, Underwood MD, Hen R, Rosoklija GB, Dwork AJ, Mann J, Arango V (2009). Antidepressants increase neural progenitor cells in the human hippocampus. Neuropsychopharmacology.

[15] Boldrini M, Santiago AN, Hen R (2013). Hippocampal granule neuron number and dentate gyrus volume in antidepressant-treated and untreated major depression. Neuropsychopharmacology.

[16] Nibuya M, Morinobu S, Duman RS (1995). Regulation of BDNF and trkB mRNA in rat brain by chronic electroconvulsive seizure and antidepressant drug treatments. J Neurosci.

[17] Russo-Neustadt AA, Beard RC, Huang YM, Cotman CW (2000). Physical activity and antidepressant treatment potentiate the expression of specific brain-derived neurotrophic factor transcripts in the rat hippocampus. Neuroscience.

[18] Chen B, Dowlatshahi D, MacQueen GM, Wang JF, Young LT (2001). Increased hippocampal BDNF immunoreactivity in subjects treated with antidepressant medication. Biol Psychiatry.

[19] Duman R, Voleti B (2012). Signaling pathways underlying the pathophysiology and treatment of depression: novel mechanisms for rapid-acting agents. Trends Neurosci.

[20] Egan MF, Kojima M, Callicott JH (2003). The BDNF val66met polymorphism affects activity-dependent secretion of BDNF and human memory and hippocampal function. Cell.

[21] Montag C, Weber B, Fliessbach K, Elger C, Reuter M (2009). The BDNF Val66Met polymorphism impacts parahippocampal and amygdala volume in healthy humans: incremental support for a genetic risk factor for depression. Psychol Med.

[22] Banasr M, Valentine GW, Li XY, Gourley S, Taylor J, Duman RS (2007). Chronic unpredictable stress decreases cell proliferation in adult cerebral cortex of rat. Biol Psychiatry.

[23] Rajkowska G, Miguel-Hidalgo JJ, Wei J (1999). Morphometric evidence for neuronal and glial prefrontal cell pathology in major depression. Biol Psychiatry.

[24] Rajkowska G, Miguel-Hidalgo JJ (2007). Gliogenesis and glial pathology in depression. CMS Neurol Disord Drug Targets.

[25] Kang H, Voleti B, Hajszan T (2012). Decreased expression of synapse-related genes and loss of synapses in major depressive disorder. Nat Med.

[26] Shansky R, Morrison JH (2009). Stress-induced dendritic remodeling in the medial prefrontal cortex: effects of circuit, hormones and rest. Brain Res.

[27] Liu RJ, Aghajanian GK (2008). Stress blunts serotonin- and hypocretin-evoked EPSCs in prefrontal cortex: role of corticosterone-mediated apical dendritic atrophy. Proc Natl Acad Sci USA.

[28] McEwen B (2012). The ever-changing brain: cellular and molecular mechanisms for the effects of stressful experiences. Dev Neurobiol.

[29] Sahay A, Hen R (2008). Hippocampal neurogenesis and depression. Novartis Found Symp.

[30] Kitayama I, Yaga T, Kayahara T, Nakano K, Murase S, Otani M, Nomura J (1997). Long-term stress degenerates, but imipramine regenerates, noradrenergic axons in the rat cerebral cortex. Biol Psychiatry.

[31] Rajkowska G (2000). Postmortem studies in mood disorders indicate altered numbers of neurons and glial cells. Biol Psychiatry.

[32] Watanabe Y, Gould E, Daniels DC, Cameron H, McEwen BS (1992). Tianeptine attenuates stress-induced morphological changes in the hippocampus. Eur J Pharmacol.

[33] Sairanen M, Lucas G, Ernfors P, Castren M, Castren E (2005). Brain-derived neurotrophic factor and antidepressant drugs have different but coordinated effects on neuronal turnover, proliferation, and survival in the adult dentate gyrus. J Neurosci.

[34] Li Y, Luikart BW, Birnbaum S (2008). TrkB regulates hippocampal neurogenesis and governs sensitivity to antidepressive treatment. Neuron.

[35] Park H, Poo MM (2013). Neurotrophin regulation of neural circuit development and function. Nat Rev Neurosci.

[36] Aid T, Kazantseva A, Piirsoo M, Palm K, Timmusk T (2007). Mouse and rat BDNF gene structure and expression revisited. J Neurosci Res.

[37] Pruunsild P, Kazantseva A, Aid T, Palm K, Timmusk T (2007). Dissecting the human BDNF locus: bidirectional transcription, complex splicing, and multiple promoters. Genomics.

[38] Lu B (2003). Pro-region of neurotrophins: role in synaptic modulation. Neuron.

[39] Sasi M, Vignoli B, Canossa M, Blum R (2017). Neurobiology of local and intercellular BDNF signaling. Pflugers Arch.

[40] Rantamäki T, Hendolin P, Kankaanpää A (2007). Pharmacologically diverse antidepressants rapidly activate brain-derived neurotrophic factor receptor TrkB and induce phospholipase-Cgamma signaling pathways in mouse brain. Neuropsychopharmacology.

[41] Saarelainen T, Hendolin P, Lucas G (2003). Activation of the TrkB neurotrophin receptor is induced by antidepressant drugs and is required for antidepressant-induced behavioral effects. J Neurosci.

[42] Kessels H, Malinow R (2009). Synaptic AMPA receptor plasticity and behavior. Neuron.

[43] Li N, Liu RJ, Dwyer J (2011). Glutamate N-methyl-d-aspartate receptor antagonists rapidly reverse behavioral and synaptic deficits caused by chronic stress exposure. Biol Psychiatry.

[44] Notaras M, Hill R, van den Buuse M (2015). The BDNF gene Val66Met polymorphism as a modifier of psychiatric disorder susceptibility: progress and controversy. Mol Psychiatry.

[45] Ninan I, Bath KG, Dagar K, Perez-Castro R, Plummer MR, Lee FS, Chao MV (2010). The BDNF Val66Met polymorphism impairs NMDA receptor-dependent synaptic plasticity in the hippocampus. J Neurosci.

[46] Pattwell SS, Bath KG, Perez-Castro R, Lee FS, Chao MV, Ninan I (2012). The BDNF Val66Met polymorphism impairs synaptic transmission and plasticity in the infralimbic medial prefrontal cortex. J Neurosci.

[47] Chen ZY, Jing D, Bath KG (2006). Genetic variant BDNF (Val66Met) polymorphism alters anxiety-related behavior. Science.

[48] Bath KG, Jing DQ, Dincheva I (2012). BDNF Val66Met impairs fluoxetine-induced enhancement of adult hippocampus plasticity. Neuropsychopharmacology.

[49] Krishnan V, Nestler EJ (2010). Linking molecules to mood: new insight into the biology of depression. Am J Psychiatry.

